# Nurse-led atrial fibrillation clinics in primary health care: a review of the evidence

**DOI:** 10.1080/02813432.2025.2466175

**Published:** 2025-02-18

**Authors:** Maria Dahlberg, Ulf Jakobsson

**Affiliations:** a Department of Clinical Sciences (Malmö), Faculty of Medicine, Center for Primary Health Care Research, Lund University, Malmö, Sweden; b University Clinic Primary Care Skåne, Region Skåne, Sweden

**Keywords:** Atrial fibrillation, literature review, nurse-led care, primary health care, cardiac arrhytmias

## Abstract

**Background:**

Atrial fibrillation (AF) is the most common arrhythmia worldwide and the majority of AF patients are treated in primary care. In order to minimize hospitalizations and visits to emergency departments, nurse-led care was introduced in secondary care and primary health care (PHC). However, even though nurse-led care was initiated in PHC almost a decade ago, and ESC guidelines recommended patient-centered integrated care including PHC for patients, there seems to be a lack of scientific evidence regarding the effects.

**Aim:**

To review the scientific literature regarding the effects of nurse-led AF clinics in PHC.

**Methods:**

A systematic review of scientific literature in Medline/Cinahl. Two reviewers independently assessed the retrieved articles.

**Results:**

Only one study was found that investigated the effectiveness of nurse-led structured AF management in PHC. The results from the study indicated positive effects; 45% reduction in all-cause mortality compared to usual care and significantly lower number of all-cause hospitalizations with nurse-led care. Several studies were found analyzing the effects of nurse-led AF-care in secondary care facilities, but only one in PHC setting. The results mainly showed that nurse-led care in AF-clinics in secondary care reduces mortality, hospitalizations and visits in emergency departments.

**Conclusions:**

Even though only one study focused on PHC, the review indicated positive effects of nurse-led care for AF patients. However, the results are only based on studies performed in inpatient care. Hence, no firm conclusion can be drawn about nurse-led AF-clinics in PHC, and more research is clearly needed in this area.

## Introduction

Atrial fibrillation (AF) is the most common arrhythmia with more than 33.5 million patients diagnosed globally [1], and is a major cardiovascular health problem [2]. One in three individuals has a lifetime risk of developing AF [3]. The disease has an incidence rate that is increasing by age [4,5]. The burden of comorbidities related to AF such as hypertension, diabetes mellitus, heart failure (HF), coronary artery disease, obesity and obstructive sleep apnea are increasing, and are identified as important modifiable risk factors for developing AF. They also have a significant impact on the progression of the disease. The comorbidities related to AF play an important role in AF-related outcomes such as stroke, HF, dementia, mortality, reduced quality of life, and hospitalization [3]. Hospitalization of AF patients is expensive, and AF has become a costly health problem [6].

The most effective way to prevent AF and atherosclerotic vascular disease is to promote a healthy lifestyle throughout life, and team-based care is an effective strategy for this preventive approach [7]. An early intervention of risk factors is found to reduce the incidence of AF. The significant morbidity and mortality associated with AF, as well as the complexity of AF, requires integrative and multidisciplinary care for these patients to manage numerous comorbidities, and focus on lifestyle modifications [3] such as weight management, blood pressure control, smoking and alcohol cessation. These modifications are examples of factors that show a positive impact on the progression of AF and could even prevent AF [8].

Patient-centered integrated management of AF patients that includes primary health care (PHC) is recommended in the ESC guidelines 2021, and has a high class of recommendation (class A). This recommendation includes patient education such as lifestyle modification, psychosocial management and strategies to promote medication adherence. It is recommended to have a multidisciplinary team and functional communication between primary and secondary care. The multidisciplinary team could contain, for example, a physician, a nurse, a dietician, physiotherapist and should also involve the patient’s family/carers. The purpose of a coordinated team is to manage the complexity of AF and to focus on individual patient needs. The patient-centered care with clear communication between different specialists is also important to optimize the healthcare system resources with the aim of reducing stroke, improving symptoms and treating comorbidities for AF patients [3]. An effective PHC system grounded in evidence-based guidelines seems to be correlated with better health among the population and reduced cost for the healthcare system [9].

In order to optimize the resources and be cost-effective, nurse-led care is used to follow up AF patients. Several studies have shown that nurse-led care of patients with AF is superior to usual care by a cardiologist regarding cardiovascular hospitalizations and mortality [6] and that structured nurse-led care appears to be an effective way to follow up with these patients [6,10–12]. Such nurse-led care for AF patients is applied in both PHC and secondary care.

In several countries, PHC has a central role in the follow-up of AF patients with the opportunity to have a multidisciplinary healthcare team with a holistic approach with the patient in focus [3]. However, the PHC role and its effect on AF care seems to be sparsely investigated, especially nurse-led care.

## Aim

To review the scientific literature regarding the effects of nurse-led AF clinics in PHC.

## Method

This review (effectiveness review) was made in line with the recommendations of the PRISMA guidelines [13], and the search was done in the Medline and Cinahl databases [14]. All searches were restricted to studies reported in English but had no time or region limit. The literature search was done both with individual terms and combinations of terms (e.g. AF, nurse-led OR nurse-led) and the setting (e.g. primary care, PHC and outpatient).

The primary outcomes in this review were patient self-management, symptom control, mortality and health service use such as rates of hospitalization and visits at emergency departments. Both standardized measures and single items were included. Secondary outcomes included economic outcomes related to the nurse-led AF clinics in PHC if data were available.

Both authors reviewed all search results (titles and abstracts) for possible inclusion, and articles selected for possible inclusion were subject to full-text assessment independently by both authors. Any discrepancies were resolved by discussion to reach consensus. An assessment of the selected studies’ quality was evaluated based on study methods, publication year, country, study design, characteristics of participants, context, sample size, response rate and outcome measures. Only the articles that met the inclusion criteria and were considered to have sufficient methodological quality were included in the review.

Due to the sparse number of studies found in this review, no explicit synthesis of the main findings was performed but two themes were structured in the presentation of the results.

## Results

The literature search initially resulted in 2830 articles (Medline) and 1298 articles (Cinahl) when searching for a combination of Atrial fibrillation AND Primary care, but when also adding nurse-led, it resulted in a further 120 articles in Medline and 11 articles in Cinahl. A total of 131 abstracts and 27 full-text articles were reviewed in this study. However, only one study was found that investigated the effectiveness of structured AF management with a specific focus on PHC [15]. The result of this randomized non-inferiority trial shows that integrated care for elderly AF patients in primary care led to a 45% (95% CI 0.37–0.82) reduction in all-cause mortality (2-year mortality) compared to usual care (follow-up by cardiologists and anticoagulation clinics). The study included 527 AF patients aged 65+ and 713 patients as controls. The integrated care contained quarterly check-ups by trained nurses in PHC. These check-ups included control of symptoms and comorbidities, control of signs of HF and patient education. The check-ups also implicated anticoagulation monitoring (kidney-function or INR-measurement) and medication compliance. The last part of the check-ups included collaboration with cardiologists and anticoagulation clinics with easy access to these specialists for assistance if needed. Additionally, the result shows that the number of all-cause hospitalizations was 16% lower in the intervention group [15].

Compared to previous research the patients in this study were, on average, 10 years older than in earlier studies from secondary care. Furthermore, the AF patients in PHC had more comorbidities [15].

Apart from the one study that had a specific focus on PHC, several studies compared nurse-led care with usual care with a focus on AF clinics in secondary care [10,11,16,17]. The outcome of the studies could be compiled into two main subcategories comparing nurse-led care versus usual care.

### Hospitalization and emergency department visits

The use of a nurse-led AF clinic in secondary care is found to reduce hospitalizations and emergency department visits [10,11,15,16]. A study [16] from the United States implied that cardiovascular disease is the leading cause of death and is the most frequent reason for hospitalization. One-third of the patients in the study who were discharged from hospital were rehospitalized within 90 days. Furthermore, many of those did not visit a clinic for a follow-up during this period. An early follow-up at a nurse-led clinic for patients with, for example, AF was associated with improved clinical outcomes such as fewer visits at emergency departments and decreased mortality [16]. Another study conducted in Denmark also had a result indicating that nurse-led care reduced the risk of acute hospitalization [18]. One study showed that emergency department visits were reduced by 82% after the implementation of nurse-led care for AF patients [11]. However, one study was found [17] that did not find a statistically significant impact of nurse-led care versus usual care regarding cardiovascular death and hospital admission. The studies mentioned above had a broader focus and did not explicitly focus on AF care and nurse-led clinics (even if AF patients were included in all studies). Thus, nurse-led clinics seem to be beneficial, not only for AF patients but also for patients with cardiovascular diseases in general.

### Mortality and adherence to guidelines

Studies in AF clinics have mainly investigated outcomes associated with mortality, healthcare utilization and quality of care. The definition of quality of care contains the function of guidelines adherence in the AF clinics, including aspects such as counseling and information about alcohol use, smoking, sleep apnea and anti-thrombotic treatment. The results show that nurse-led care in AF clinics is a better-quality model compared to usual care [11]. One study reports guidelines adherence in nurse-led care where 96% of the patients received treatment according to the guidelines compared to usual care where 70% of the patients got evidence-based care. In this study, nurse-led care versus usual care also showed improved mental health and vitality in the intervention group. Nurse-led care specifically after catheter ablation showed significant improvements regarding tiredness, headache, concentration difficulties and sleeping issues compared to usual care [11].

A study from the Netherlands compared nurse-led care versus usual care for patients with AF. The results showed that nurse-led care was superior to usual care. Adherence to guideline recommendations was significantly better in the nurse-led care and these patients were also better informed about their disease. Additionally, cardiovascular mortality and hospitalizations decreased in the nurse-led care [6]. Another study from the Netherlands [17] showed that nurse-led care versus usual care was superior regarding guidelines-based recommendations for these patients with first-detected AF in secondary care. The guideline-based recommendations that included diagnostic and treatment procedures were 61% with nurse-led care compared to 26% with usual care. These procedures maintained, for example, monitoring blood pressure, renal function and glucose intolerance.

In summary, the studies regarding nurse-led care of AF patients in secondary care show a clear indication of a reduction in mortality and cardiovascular hospitalization [6,10,15,16], to the same costs as for usual care [19].

## Discussion

Only one study was found that focused on nurse-led AF clinics in a PHC context. Thus, there is a great need for more research to evaluate the effect of AF clinics in PHC. However, despite the lack of evidence regarding the PHC context, several studies were found focusing on nurse-led AF clinics. This research focusing on nurse-led care shows positive effects on numerous variables such as mortality, medication adherence, emergency department visits, hospitalization and quality of care [6,10,11,15], indicating that such AF clinics most likely improve the quality of care regardless of context. Only one study did not support the positive effect of nurse-led care versus usual care regarding reducing the risk of cardiovascular death and hospital admission [17].

The outcome of this study mainly supports the notion of improved effect of nurse-led care for AF patients when compared to conventional care. For example, a 45% reduced mortality risk and 16% lower risk for hospitalization were found [15]. Previous research comparing nurse-led clinics with conventional hospital care has shown similar results [6]. Furthermore, nurse-led clinics have been found to be more cost-effective, have significantly shorter waiting times, and lead to increased patient medication adherence [11]. However, only one study has investigated the effects of nurse-led care specifically in PHC. On the one hand, this study clearly showed the positive effects of nurse-led AF clinics in PHC, and had several strengths such as randomization, large sample size, and included a wide range of variables at baseline. On the other hand, the external validity can be seen to be limited since the data collection was only conducted in the Netherlands. Thus, the results may only be generalized to the Netherlands and countries with similar context (e.g. European countries). Another limitation of the study was that information about echocardiography, NT-proBNP and type of AF (paroxysmal, persistent or permanent) could make the interpretation of the results more difficult, especially from a clinical point of view. Further research is thus needed to investigate the impact of nurse-led AF care in PHC. According to this review, the PHC context seems to be unexplored territory in this matter.

It is recommended in the ESC guidelines to have integrated care of AF patients including, for example, patient education, lifestyle modifications and multidisciplinary teams [3]. The studies that were found focused on secondary care and did not reveal if this task primarily is for PHC or AF clinics in secondary care, and if an early structured nurse-led follow-up in primary care could affect the frequency of hospitalization.

The ESC guidelines recommend a structured follow-up and better communication between primary and secondary care in order to optimize the use of healthcare resources [3]. However, even if such cooperation between primary and secondary care is most likely beneficial in the care of AF patients, this does not seem to have been evaluated yet in any research study. Such studies should focus on the quality of the present cooperation as well as methods to optimize such communication/cooperation.

Primary care’s function is to have responsibility for the follow-up of AF patients among other patient categories. Studies show a difference between the AF-patient category in primary and secondary care. In primary care, the patients seem to be older and suffer more often from comorbidities, which indicates a more complex and resource-demanding situation for PHC. Both patient categories seem to have a reduced risk of cardiovascular hospitalization and death with nurse-led care [6]. The typical patient in primary care generally has multiple diseases and not merely AF. These other comorbidities need to be managed together and integrated with AF care to prevent stroke and hospitalization and to improve the quality of life for these patients. This complexity is one of the motives for integrated care being needed to manage the increasing burden of AF [15]. Thus, primary care has an important role in the follow-up of AF patients, especially the nurse-led clinics. However, since there seems to be a lack of scientific literature focusing on AF patients in out patient care, more research is needed.
